# Microdiscectomy versus autologous conditioned serum for degenerative lumbar disc disease with radiculopathy: a 12–month exploratory study

**DOI:** 10.3389/fneur.2026.1972710

**Published:** 2026-10-20

**Authors:** Paweł Gogol, Rafał Wiśniewski, Rafał Pasztaleniec, Robert Szczepaniak, Michał Sobstyl, Rafał Staszkiewicz, Paweł Ratuski, Beniamin Oskar Grabarek

**Affiliations:** 1 Neurolocus Pain Management Centre, Warsaw, Poland; 2 Pain Treatment Clinic, Our Lady of Perpetual Help Hospital in Wołomin, Wołomin, Poland; 3 Department of Neurosurgery, Faculty of Medicine, Academy of Silesia, Katowice, Poland; 4 Pain Medicine Centre, Piła, Poland; 5 Department of Neurosurgery, Institute of Psychiatry and Neurology, Warsaw, Poland; 6 Department of Neurosurgery, 5th Military Clinical Hospital with the SP ZOZ Polyclinic in Kraków, Kraków, Poland; 7 Department of Rehabilitation, Gruca Orthopedic and Trauma Teaching Hospital in Otwock, Centre of Postgraduate Medical Education, Otwock, Poland; 8 Collegium Medicum, WSB University, Dąbrowa Górnicza, Poland

**Keywords:** autologous conditioned serum, fatigue, lumbar disc herniation, microdiscectomy, Orthokine, quality of life, radiculopathy, sexual satisfaction

## Abstract

**Inroduction:**

Lumbar degenerative disc disease with radiculopathy that fails conservative care is usually treated surgically. Autologous conditioned serum (Orthokine^®^), an injectable anti-inflammatory blood-derived product, offers a less invasive alternative. Comparisons of these treatments rarely look beyond pain and disability, so quality of life, fatigue, and sexual functioning remain largely unexplored. We compared microdiscectomy and autologous conditioned serum across these domains and structural imaging outcomes over 12 months.

**Methods:**

In this prospective, non-randomised cohort study, 140 patients with radiculopathy unresponsive to at least 6 months of conservative treatment underwent microdiscectomy (n = 86) or autologous conditioned serum injections (n = 54). Herniation morphology guided the clinical recommendation, and the final choice was made jointly by physician and patient. We assessed pain intensity, disability, pain quality, health-related quality of life, life satisfaction, fatigue, sexual satisfaction (Davies Sexual Satisfaction Scale), serum C-reactive protein, and magnetic resonance imaging parameters at baseline and at 3, 6, and 12 months. These outcomes were analysed with mixed-design (group × time) analysis of variance.

**Results:**

Both treatments produced large improvements in every patient-reported domain, sustained to 12 months. Microdiscectomy provided faster relief. Significant group × time interactions for pain, disability, pain quality, physical and mental quality of life, and sexual satisfaction reflected advantages for microdiscectomy at 3 and partly 6 months, which were no longer significant at 12 months. The early differences in pain and disability persisted after adjustment for age, baseline scores, and herniation type. Life satisfaction and fatigue improved in parallel in both groups. Sexual satisfaction rose to near-maximal levels, and C-reactive protein declined similarly, in both groups. Spinal canal occupation remained lower after microdiscectomy, whereas disc height increased more after autologous conditioned serum from 6 months onward.

**Discussion:**

At 12 months, the two treatments achieved comparable outcomes for pain, disability, quality of life, fatigue, and sexual satisfaction. Surgery offered quicker relief, whereas the injection-based approach was less invasive and was associated with greater disc-height gain. Measuring sexual satisfaction and fatigue alongside conventional endpoints broadens the evidence available for shared treatment decisions. These exploratory findings warrant confirmation in randomised trials.

## 1 Introduction

Lumbar disc herniation and degenerative disc disease (DDD) remain among the most common musculoskeletal disorders in spine practise, affecting approximately one in ten adults over their lifetime and constituting a leading cause of work absence and chronic disability worldwide (1, 2). Beyond radicular pain and functional impairment, the condition carries a substantial and often under–recognised burden on broader domains of well–being, including health–related quality of life (HRQoL), fatigue, mood, and sexual functioning (3–5). Most comparative studies of lumbar DDD treatments, however, rely predominantly on pain and disability endpoints, leaving these patient–centred dimensions under–explored.

When conservative management fails, two broad pathways are considered: surgical decompression, most often by microdiscectomy, and biologically oriented, minimally invasive alternatives. Microdiscectomy achieves direct mechanical decompression and is supported by extensive evidence, including recent meta–analyses of minimally invasive variants (6–8). An alternative is autologous conditioned serum (ACS, Orthokine^®^), a blood–derived preparation enriched in interleukin–1 receptor antagonist (IL–1Ra) and other anti–inflammatory mediators, thought to counteract the IL–1–driven cascade implicated in disc degeneration and nerve–root sensitisation (9, 10). Evidence for ACS in lumbar radiculopathy has accumulated over two decades, from early controlled comparisons with corticosteroid injections (9) and prospective case series (11), to randomised, placebo–controlled trials targeting the dorsal root ganglion (9, 12), with growing interest in orthobiologic strategies such as platelet–rich plasma (13, 14).

Sexual dysfunction affects a large proportion of patients with chronic low back pain, with near–complete cessation of sexual activity described in some cohorts (4, 5), and recent reviews confirm that sexual difficulties are common, multidimensional, and closely linked to pain, disability, mood, and relationship factors (4, 5, 15, 16). Fatigue is likewise present in most patients on long–term sick leave for low back pain and independently predicts disability (17). Despite this, validated sexual–satisfaction and fatigue instruments are rarely used in comparative DDD studies, and, to our knowledge, no previous study has directly compared microdiscectomy and ACS using a multidimensional sexual–satisfaction instrument. Understanding whether a minimally invasive biological therapy can match the clinical benefit of surgical decompression is directly relevant to everyday treatment decisions for patients with lumbar DDD and radiculopathy who have failed conservative care. Current comparative evidence focuses almost exclusively on pain and disability, leaving clinicians without guidance on how the two pathways compare across quality of life, fatigue, and sexual functioning—domains that strongly influence patients’ overall satisfaction with treatment and their willingness to accept a more invasive procedure. By assessing these outcomes together with structural MRI parameters over a full 12–month course, this study aims to provide clinically actionable evidence to support shared decision–making between microdiscectomy and ACS injections.

The aim of this study was to compare microdiscectomy and ACS injections in patients with lumbar DDD and radiculopathy refractory to conservative treatment, across pain, disability, quality of life, fatigue, sexual satisfaction, and MRI structural parameters, over 12 months.

## 2 Methods

### 2.1 Study design and population

We conducted a prospective, non–randomised comparative cohort study of patients with symptomatic lumbar DDD and radiculopathy unresponsive to ≥6 months of conservative management. Treatment assignment (microdiscectomy vs. ACS) followed shared decision–making based on herniation morphology and patient preference.

For every patient, the treating physician reviewed the baseline MRI and discussed the diagnosis and both treatment pathways in detail. Patients were informed that microdiscectomy is reimbursed under the Polish National Health Fund (NFZ), whereas ACS (Orthokine^®^) is not reimbursed and is financed entirely out–of–pocket. Herniation morphology guided the clinical recommendation (extrusion/sequestration favouring microdiscectomy; contained protrusion favouring ACS), and the final treatment was chosen jointly by the physician and patient after weighing invasiveness, expected recovery, and patient preference. Importantly, in no instance did a patient for whom ACS was the clinically recommended option instead undergo microdiscectomy for financial reasons; the funding difference was disclosed for informed consent but did not override the shared clinical decision.

In total, 140 patients were enrolled: 86 microdiscectomy, 54 ACS (mean age 48.7 ± 8.2 years, range 24–60; 86 men, 54 women), all with complete follow–up at 3, 6, and 12 months. Reporting follows the STROBE recommendations for observational studies (18). The study was approved by the Bioethics Committee of the Regional Medical Chamber in Kraków (decision 6/KBL/OIL/2025 of March 11, 2025) and was carried out in line with the Declaration of Helsinki.

### 2.2 Eligibility criteria

Common inclusion criteria were written informed consent; MRI–confirmed lumbar DDD (≥1.5 T scanner); self–reported White (Caucasian) ethnicity; age 18–60 years; baseline VAS/NRS ≥ 4; and no improvement after ≥6 months of conservative treatment. Microdiscectomy candidates additionally required no internal–medicine contraindications to surgery, ability to discontinue anticoagulant/analgesic medication, and absence of a rapidly progressive neurological deficit; ACS candidates required ability to complete the full injection course and the possibility of discontinuing anticoagulant (after consultation with the treating physician) and analgesic medication for 4 weeks before baseline assessment. Exclusion criteria were active malignancy, autoimmune disease, pregnancy/lactation, prior lumbar spine surgery, scoliosis, sacroiliac joint arthritis/arthropathy, and active infection.

Radiculopathy severity and neurological status were assessed clinically at baseline by the treating physician, incorporating radicular pain intensity (NRS), the straight-leg-raise (Lasègue) sign, dermatomal sensory testing, manual muscle testing (Medical Research Council [MRC] scale), and deep tendon reflexes; overall radiculopathy severity was graded on a composite 0–3 scale (absent, mild, moderate, severe) based on this assessment.

### 2.3 Interventions

#### 2.3.1 Microdiscectomy

Patients allocated to surgery underwent standard lumbar microdiscectomy under general anaesthesia using microscopic or loupe magnification through a limited posterior mini-open approach, with partial medial facetectomy performed where required for adequate exposure and decompression. The compressed nerve root was decompressed by removal of the herniated or sequestrated disc fragment, preserving uninvolved disc structures where feasible. Postoperative care followed a standardised enhanced–recovery protocol with early mobilisation.

#### 2.3.2 Autologous conditioned serum (ACS, Orthokine^®^)

Patients assigned to the ACS group underwent preparation of autologous conditioned serum (Orthokine^®^) per the manufacturer’s standardised protocol: venous blood was collected into syringes containing chromium–treated glass beads and incubated at 37 °C before centrifugation to obtain serum enriched in interleukin–1 receptor antagonist and other anti–inflammatory mediators. All patients received four image-guided (fluoroscopy or ultrasound) periradicular epidural injections of ACS, 4 mL per session, at approximately 7-day intervals (days 0, 7, 14 and 21), delivered at the level of the affected disc and at the level immediately below it.

### 2.4 Follow–up schedule

Clinical, patient–reported, and radiological assessments were performed at four pre–specified time points: before treatment (T0) and at 3, 6, and 12 months. All 140 patients had complete data at every time point. Validated Polish–language versions of all questionnaires were used.

### 2.5 Patient–reported outcome measures

All patients completed the same battery of instruments at T0, T3, T6, and T12.

#### 2.5.1 Pain intensity: Visual Analogue Scale and Numerical Rating Scale

Pain intensity was captured with two complementary single–item measures. The VAS is a 10 cm horizontal line anchored by “no pain” and “worst pain imaginable,” scored 0–10 from the distance marked; the NRS asks patients to select a whole number from 0 to 10. Both are widely used and well validated for tracking within–patient change in musculoskeletal pain, with higher scores indicating more severe pain (19–21).

#### 2.5.2 Disability: Oswestry Disability Index

Disability was assessed with the Oswestry Disability Index, a ten–section questionnaire covering pain and daily functioning (personal care, lifting, walking, sitting, standing, sleeping, sexual activity, social life, travelling), each scored 0–5; the sum is expressed as a percentage (0–100%), with higher scores indicating greater disability. The ODI is a standard, extensively validated outcome measure in lumbar spine research (22, 23).

#### 2.5.3 Pain quality: Short–Form McGill Pain Questionnaire

Pain quality was assessed with the Short–Form McGill Pain Questionnaire: 15 descriptors (11 sensory, 4 affective), each rated 0 (none) to 3 (severe), yielding sensory, affective, and total scores plus a 0–5 Present Pain Intensity (PPI) index; higher scores indicate more intense, more distressing pain (24).

#### 2.5.4 Health–related quality of life: 36–item Short–Form Health Survey

Generic HRQoL was assessed with the SF–36, whose eight domains combine into a Physical Component Summary (PCS) and Mental Component Summary (MCS), each scored 0–100 with higher values indicating better health status (25).

#### 2.5.5 Life satisfaction: Satisfaction With Life Scale

Global life satisfaction was measured with the Satisfaction With Life Scale, a 5–item instrument (7–point Likert scale) summed to a total score of 5–35, with higher scores reflecting greater life satisfaction (26).

#### 2.5.6 Fatigue: Functional Assessment of Chronic Illness Therapy–Fatigue

Fatigue was assessed with the FACIT–Fatigue subscale, a 13–item instrument originally developed for oncology and validated across chronic illnesses. Items are rated 0–4 over the preceding 7 days; after reverse–scoring, the total ranges from 0 to 52, with higher scores indicating less fatigue (27).

#### 2.5.7 Sexual satisfaction: Davies Sexual Satisfaction Scale

Sexual satisfaction was assessed with the Davies Sexual Satisfaction Scale (DSSS), a 21–item, patient–reported instrument (5–point Likert scale) (28), yielding three subscales—Physical (11 items), Emotional (4 items), and Control (6 items)—summing to a total score of 21–105, with higher scores indicating greater sexual satisfaction. A small, balanced proportion of item responses were missing; subscale and total scores were computed as the mean of answered items multiplied by the number of items, consistent with the source database’s pro–rating approach.

### 2.6 Inflammatory marker (CRP)

Serum C–reactive protein (CRP) concentrations were measured at baseline (T0) and at the 3–, 6–, and 12–month follow–up visits (T3, T6, and T12, respectively) in both treatment groups. Venous blood was collected into serum–separator tubes and processed according to the laboratory’s standard operating procedures. All measurements were performed in the same accredited clinical laboratory using consistent analytical procedures and internal quality–control protocols. CRP was treated as an acute phase reactant, which was a nonspecific marker of systemic inflammation. CRP was not considered a direct measure of local intervertebral disc inflammation or IL–1 pathway activity.

### 2.7 Radiological assessment

At every time point, patients underwent lumbosacral MRI on a ≥ 1.5 T scanner, assessing Pfirrmann grade (1–5), Modic changes (0–III), high–intensity zone (present/absent), nerve–root compression (0–3), Fujiwara–Weishaupt canal stenosis grade (0–3), percentage spinal–canal occupation, and intervertebral disc height (mm) (29).

Baseline MRI scans were independently evaluated by three spine specialists (R.St., P.G., R.W.) before the treatment decision was finalised; the readers were therefore blinded to the subsequently selected treatment pathway. Inter–rater reliability was assessed across the 140 baseline MRI examinations. Agreement was excellent for the continuous parameters, assessed with the intraclass correlation coefficient (ICC[2,k]): disc height, ICC = 0.95 (95% CI 0.93–0.97); percentage spinal–canal occupation, ICC = 0.94 (95% CI 0.91–0.96). For the ordinal parameters, assessed using the mean pairwise linearly weighted Cohen’s kappa across the three readers: Pfirrmann grade, κ = 0.89 (95% CI 0.67–0.94); Modic changes, κ = 0.88 (95% CI 0.82–0.93); nerve–root compression, κ = 0.90 (95% CI 0.86–0.95); Fujiwara–Weishaupt grade, κ = 0.89 (95% CI 0.83–0.94). For the categorical parameter (high–intensity zone, present/absent), agreement was assessed with Fleiss’ κ = 0.91 (95% CI 0.86–0.96). Discordant ratings were resolved by consensus, with the final radiological interpretation used in clinical decision–making assigned to the treating physician of record. Follow–up MRI at 3, 6, and 12 months was read by the same panel; because post–treatment images can show features consistent with prior surgery, full blinding to the treatment received could not be maintained beyond the baseline reliability assessment.

### 2.8 Statistical analysis

Continuous variables are presented as mean ± SD; categorical variables as counts and percentages. Normality was assessed with the Shapiro–Wilk test. Between–group comparisons used the independent–samples *t*-test (Welch–corrected where variances were unequal) or the Mann–Whitney U test where normality was not met; categorical variables were compared with the chi–squared or Fisher’s exact test (expected cell counts < 5).

Outcome evolution over time (T0–T12) and between–group differences were analysed with two–way mixed–design ANOVA (group × time), reporting main effects, the interaction term, and η^2^p, with Greenhouse–Geisser correction where indicated. Within–group change from baseline used the paired *t*-test or Wilcoxon test (effect size dz); between–group comparisons at each time point used the same parametric/non–parametric approach as for baseline variables (Cohen’s d). Ordinal MRI variables were analysed within groups with the Friedman test and between groups with the Mann–Whitney U test; Modic distribution used the chi–squared test. As a sensitivity analysis, given the baseline imbalances in Section 4.1, principal pain and disability outcomes at 3 and 12 months were re–examined with ANCOVA adjusting for baseline value, age, and herniation type. Serum CRP was analysed over time using the same mixed–design (group × time) ANOVA approach as the principal patient–reported outcomes. As a secondary, hypothesis–generating analysis motivated by known sex differences in the mechanisms of sexual dysfunction, DSSS total scores were additionally examined by sex (male vs. female) within each treatment group using mixed–design (sex × time) ANOVA (Supplementary materials). Significance was set at two–sided *p* < 0.05; significant results are marked with an asterisk (*) in tables. Analyses were performed in STAT Plus software (AnalystSoft, Brandon, FL33511, United States).

## 3 Results

### 3.1 Baseline characteristics

Baseline demographic, clinical, and MRI characteristics are shown in Table 1. Patients in the microdiscectomy group were older (50.4 ± 7.5 vs. 46.1 ± 8.6 years; *p* = 0.004); BMI, symptom duration, prior conservative treatment duration, baseline Pfirrmann grade, canal occupation, and disc height did not differ significantly.

**Table 1:** Baseline demographic and clinical characteristics of the two treatment groups.

Characteristic/category	Microdiscectomy (*n* = 86)	ACS (*n* = 54)	Test	*p*
Age (years)	50.38 ± 7.48	46.09 ± 8.59	Mann–Whitney U	0.004*
BMI (kg/m^2^)	26.96 ± 2.86	26.83 ± 3.27	Mann–Whitney U	0.665
Symptom duration (months)	47.74 ± 23.02	49.24 ± 24.39	Mann–Whitney U	0.630
Prior conservative treatment (months)	13.76 ± 5.47	13.94 ± 5.66	Mann–Whitney U	0.935
Baseline Pfirrmann grade (1–5)	4.10 ± 0.49	4.13 ± 0.52	Mann–Whitney U	0.760
Baseline spinal canal occupation (%)	56.13 ± 9.53	55.84 ± 9.40	Welch t-test	0.861
Baseline disc height (mm)	7.93 ± 0.96	7.93 ± 0.89	Welch t-test	0.993
Sex				0.811
Female	32 (37.2%)	22 (40.7%)		
Male	54 (62.8%)	32 (59.3%)		
Treated level				0.430
L3/L4	7 (8.1%)	3 (5.6%)		
L4/L5	43 (50.0%)	33 (61.1%)		
L5/S1	36 (41.9%)	18 (33.3%)		
Herniation type				<0.001*
Extrusion	47 (54.7%)	17 (31.5%)		
Protrusion	21 (24.4%)	33 (61.1%)		
Sequestration	18 (20.9%)	4 (7.4%)		
Baseline Modic changes				0.445
None	12 (14.0%)	12 (22.2%)		
Modic I	37 (43.0%)	24 (44.4%)		
Modic II	35 (40.7%)	16 (29.6%)		
Modic III	2 (2.3%)	2 (3.7%)		
Radiculopathy severity				0.343
Moderate	45 (52.3%)	23 (42.6%)		
Severe	41 (47.7%)	31 (57.4%)		
Motor deficit present	9 (10.5%)	6 (11.1%)	Fisher’s exact	1.000
BMI category				0.899
Normal	21 (24.4%)	15 (27.8%)		
Overweight	54 (62.8%)	32 (59.3%)		
Obese	11 (12.8%)	7 (13.0%)		
Smoking status				0.825
Current	18 (20.9%)	11 (20.4%)		
Former	16 (18.6%)	8 (14.8%)		
Never	52 (60.5%)	35 (64.8%)		
Comorbidities				
Controlled hypertension	17 (19.8%)	12 (22.2%)	Fisher’s exact	0.831
Dyslipidemia	19 (22.1%)	14 (25.9%)	Fisher’s exact	0.684
Stable hypothyroidism	3 (3.5%)	1 (1.9%)	Fisher’s exact	1.000
Stable depression (treated)	5 (5.8%)	3 (5.6%)	Fisher’s exact	1.000

Values are mean ± SD or *n* (%). ACS, autologous conditioned serum; SD, standard deviation. Test: for continuous variables, the independent–samples *t*-test (Welch–corrected) was used where the Shapiro–Wilk test indicated an approximately normal distribution in both groups, and the Mann–Whitney U test otherwise; for categorical variables, group differences were assessed with the chi-squared or Fisher’s exact test where expected cell counts were <5 (test not shown for brevity). * *p* < 0.05.

Herniation type differed significantly between groups (*p* < 0.001): extrusions and sequestrations were more frequent with microdiscectomy (54.7 and 20.9%) and protrusions more frequent with ACS (61.1%). No other categorical baseline variable differed significantly (all *p* > 0.05). Radiculopathy severity (moderate/severe) and the presence of a motor deficit likewise did not differ significantly between groups (chi-squared *p* = 0.343 and Fisher’s exact *p* = 1.000, respectively).

### 3.2 Clinical, quality–of–life, and psychosocial outcomes over time

Across all principal patient–reported outcomes, mixed–design ANOVA showed a significant main effect of time (all *p* < 0.001, η^2^p = 0.78–0.94), with within–group effect sizes at 12 months of dz. = 1.8–4.8 (Wilcoxon/paired t, all *p* < 0.001; Table 2). A significant group × time interaction was present for pain intensity, disability, pain quality, both SF–36 component scores, and DSSS total score (Table 2), and for all three DSSS subscales (Supplementary Table S2), but not for life satisfaction or fatigue (Table 2).

**Table 2:** Summary of mixed–design ANOVA results (group × time) for the principal patient–reported outcomes, with between–group *p*-values at each follow–up time point.

Outcome	Time effect *F* (*p*, η^2^p)	Group effect *F* (*p*, η^2^p)	Group × Time *F* (*p*, η^2^p)	Between–group *p* at T3/T6/T12
Pain intensity (VAS, 0–10)	2226.8 (<0.001, 0.94)	2.47 (0.118, 0.02)	5.94 (<0.001, 0.04)	<0.001 */0.023 */0.810
Pain intensity (NRS, 0–10)	1699.2 (<0.001, 0.92)	3.82 (0.053, 0.03)	4.54 (0.004, 0.03)	<0.001 */0.010 */0.674
Disability (ODI, %)	1624.1 (<0.001, 0.92)	2.33 (0.129, 0.02)	4.64 (0.003, 0.03)	0.002 */0.179/0.616
Pain quality (SF–MPQ total)	1830.5 (<0.001, 0.93)	2.07 (0.153, 0.01)	3.97 (0.008, 0.03)	0.003 */0.016 */0.736
HRQoL – SF–36 Physical Component	1787.4 (<0.001, 0.93)	4.50 (0.036, 0.03)	4.14 (0.007, 0.03)	<0.001 */0.071/0.694
HRQoL – SF–36 Mental Component	1436.4 (<0.001, 0.91)	0.95 (0.332, 0.01)	3.64 (0.013, 0.03)	0.037 */0.312/0.422
Life satisfaction (SWLS)	481.5 (<0.001, 0.78)	2.49 (0.117, 0.02)	1.20 (0.308, 0.01)	0.096/0.074/0.919
Fatigue (FACIT–F)	1035.0 (<0.001, 0.88)	0.62 (0.433, 0.00)	1.96 (0.120, 0.01)	0.040 */0.464/0.959
Sexual satisfaction (DSSS total)	767.1 (<0.001, 0.85)	0.35 (0.556, 0.00)	7.22 (<0.001, 0.05)	0.007 */0.421/0.084

η^2^p = partial eta–squared. * *p* < 0.05.

#### 3.2.1 Pain intensity and disability

Baseline VAS and NRS did not differ between groups (*p* = 0.519 and *p* = 0.757). At 3 months, microdiscectomy had lower VAS (3.26 ± 1.03 vs. 3.92 ± 0.92; *p* < 0.001; d = −0.67) and NRS (*p* < 0.001; d = −0.61) than ACS. The difference remained significant at 6 months (VAS *p* = 0.023; NRS *p* = 0.010) and was no longer significant at 12 months (VAS *p* = 0.810; NRS *p* = 0.675; Figure 1).

**Figure 1:**
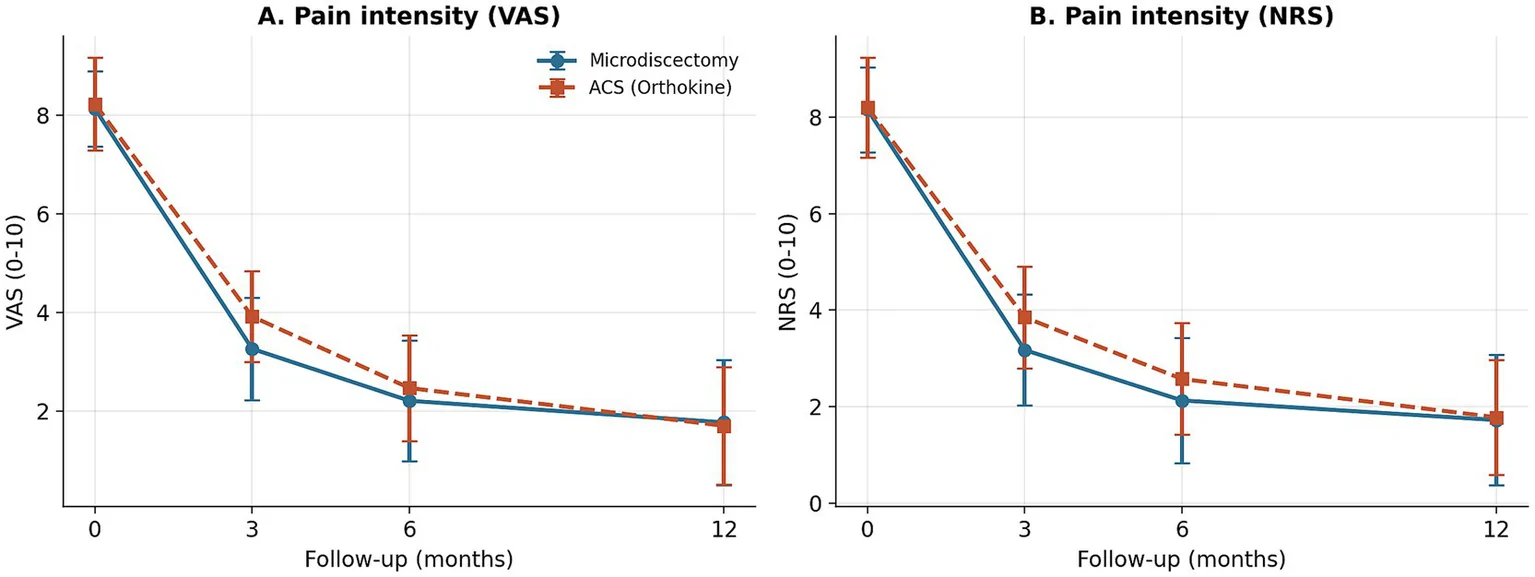
Pain intensity over time by treatment group. **(A)** Visual Analogue Scale (VAS); **(B)** Numerical Rating Scale (NRS). Points show group means; error bars show ± 1 SD.

ODI did not differ at baseline (75.7% vs. 75.1%, *p* = 0.715), was lower with microdiscectomy at 3 months (37.7% vs. 43.0%; *p* = 0.002; d = −0.61), and did not differ significantly at 6 or 12 months (*p* = 0.179 and *p* = 0.616). SF–MPQ total scores followed the same pattern, differing significantly only at 3 months (14.71 ± 4.78 vs. 17.13 ± 4.76; *p* = 0.003; d = −0.51) and 6 months (*p* = 0.016), with no difference at 12 months (*p* = 0.736; Supplementary Figure S1).

#### 3.2.2 Health–related quality of life

SF–36 PCS did not differ at baseline (*p* = 0.142), was higher with microdiscectomy at 3 months (65.64 ± 8.55 vs. 60.29 ± 8.92; *p* < 0.001; d = 0.61), and did not differ significantly thereafter (6 months *p* = 0.071; 12 months *p* = 0.694); PCS was the only outcome with a significant overall main effect of group (*p* = 0.036; Table 2). SF–36 MCS did not differ at baseline, was higher with microdiscectomy at 3 months (*p* = 0.037), and did not differ at 6 or 12 months (Supplementary Figure S2; Supplementary Table S1).

#### 3.2.3 Life satisfaction and fatigue

SWLS and FACIT–F showed no significant group × time interaction (*p* = 0.308 and *p* = 0.120; Table 2). No between–group difference reached significance for SWLS at any time point (all *p* ≥ 0.074). For FACIT–F, a difference was significant only at 3 months (32.52 ± 4.42 vs. 30.85 ± 4.76; *p* = 0.040), not at 6 or 12 months (*p* = 0.464, *p* = 0.959; Supplementary Figure S3).

#### 3.2.4 Sexual satisfaction (DSSS)—total score and subscales

At baseline, the total DSSS score was slightly lower in the microdiscectomy group than in the ACS group (49.13 ± 7.15 vs. 51.58 ± 6.86; *p* = 0.046), although the magnitude of the difference was small. At 3 months, microdiscectomy had a higher total score (73.46 ± 7.31 vs. 69.82 ± 7.89; *p* = 0.007); the difference was not significant at 6 months (*p* = 0.421) or 12 months (*p* = 0.084). The group × time interaction for total DSSS was significant (*p* < 0.001; Table 2; Supplementary Figure S3).

All three DSSS subscales showed a significant group × time interaction (Physical *p* < 0.001; Emotional *p* = 0.002; Control *p* = 0.030; full data in Supplementary Table S2). The Physical subscale was higher with microdiscectomy at 3 months (*p* = 0.009) and higher with ACS at 12 months (*p* = 0.024). The Emotional subscale was lower with microdiscectomy at baseline (*p* = 0.015), higher with microdiscectomy at 3 months (*p* = 0.033), and higher with ACS at 12 months (*p* = 0.055, borderline). The Control subscale was higher with microdiscectomy only at 3 months (*p* = 0.027). In the sex–stratified analysis, DSSS total scores improved similarly for men and women after microdiscectomy (no significant sex effect or sex × time interaction, both *p* ≥ 0.17). After ACS, men showed a numerically greater improvement than women, with a borderline sex × time interaction (*p* = 0.072) and significantly higher scores in men at 12 months (85.07 ± 7.68 vs. 80.16 ± 7.04; *p* = 0.019; Supplementary Table S3).

#### 3.2.5 Sensitivity analysis

When principal pain and disability outcomes at 3 and 12 months were re–examined with ANCOVA (adjusting for baseline value, age, and herniation type), between–group differences remained significant at 3 months for VAS, NRS, and ODI (all adjusted *p* ≤ 0.005) and were not significant at 12 months for any of these outcomes (all adjusted *p* ≥ 0.65; full results in Supplementary Table S4).

### 3.3 Inflammatory marker (CRP)

Serum CRP declined significantly over time in both groups (main effect of time: *F*(3, 414) = 139.0, *p* < 0.001, η^2^p = 0.50), with no significant group × time interaction (*F*(3, 414) = 1.59, *p* = 0.192, η^2^p = 0.011) and no significant between–group difference at any time point (all *p* ≥ 0.47; Table 3). Within–group reductions from baseline to 12 months were significant in both groups (microdiscectomy: 2.65 ± 1.44 to 1.92 ± 1.11 mg/L, dz. = 1.26; ACS: 2.67 ± 1.31 to 1.87 ± 0.98 mg/L, dz. = 1.44; both *p* < 0.001), consistent with a parallel decline in systemic inflammatory activity irrespective of treatment modality, rather than an ACS–specific anti–inflammatory signal detectable at the level of a single systemic biomarker.

**Table 3:** Serum C–reactive protein (CRP, mg/L; mean ± SD) over time by treatment group.

Time point	Microdiscectomy (*n* = 86)	ACS (*n* = 54)	p*
T0	2.65 ± 1.44	2.67 ± 1.31	0.961
T3	2.00 ± 1.15	2.14 ± 1.10	0.470
T6	1.92 ± 1.09	1.93 ± 0.99	0.956
T12	1.92 ± 1.11	1.87 ± 0.98	0.815

Values are mean ± SD. * Between–group comparison at each time point (Welch *t*-test); none reached significance. Mixed–design (group × time) ANOVA: main effect of time *F*(3, 414) = 139.0, *p* < 0.001, η^2^p = 0.50; main effect of group *F*(1, 138) = 0.02, *p* = 0.877, η^2^p < 0.001; group × time interaction *F*(3, 414) = 1.59, *p* = 0.192, η^2^p = 0.011. Within–group change from baseline to 12 months was significant in both groups (paired *t*-test, both *p* < 0.001; dz = 1.26 microdiscectomy, dz = 1.44 ACS).

### 3.4 Radiological (MRI) findings

Spinal canal occupation did not differ at baseline (56.1% vs. 55.8%; *p* = 0.861) but was lower with microdiscectomy at 3 months (26.7% vs. 30.9%; *p* < 0.001; d = −0.58), 6 months (*p* = 0.005), and 12 months (*p* = 0.031). Disc height did not differ at baseline or 3 months but was greater with ACS at 6 months (8.02 ± 0.97 vs. 8.39 ± 0.90 mm; *p* = 0.025; d = −0.39) and 12 months (*p* = 0.025; d = −0.39). The group × time interaction was significant for both canal occupation (*p* = 0.002) and disc height (*p* < 0.001, η^2^p = 0.292—the largest of any variable examined; Figure 2).

**Figure 2:**
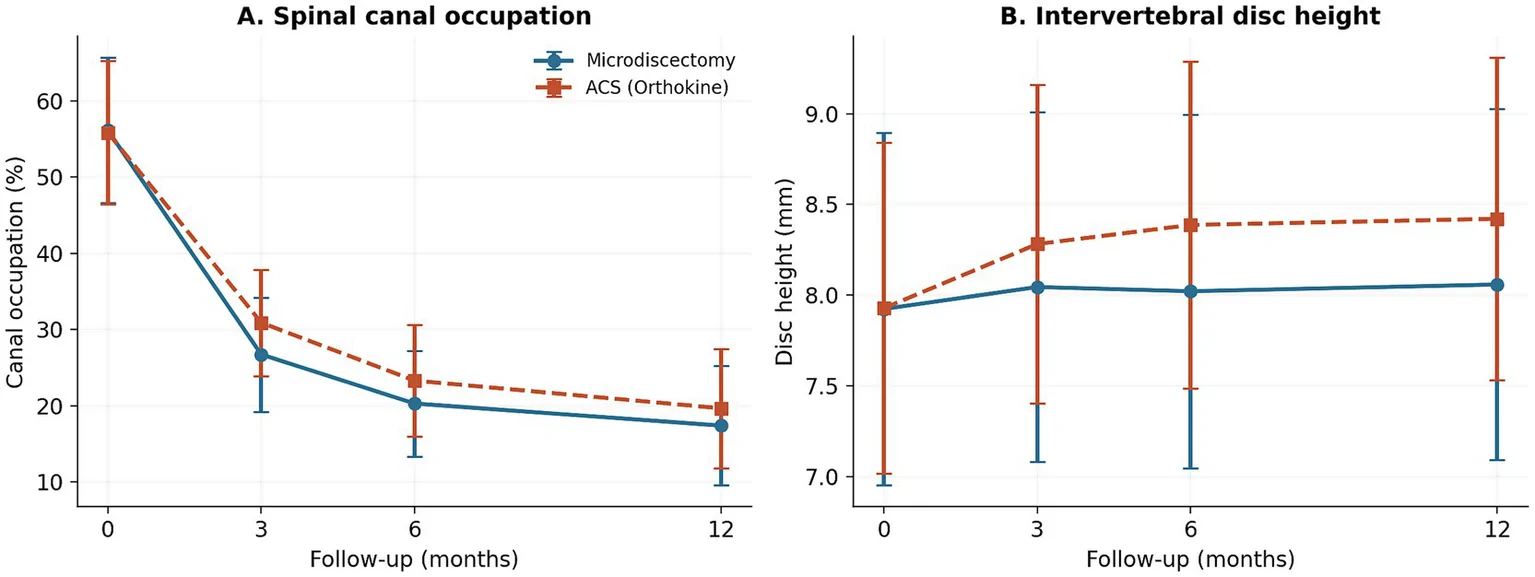
MRI structural outcomes over time by treatment group. **(A)** Spinal canal occupation (%); **(B)** intervertebral disc height (mm). Points show group means; error bars show ± 1 SD.

Pfirrmann grade changed only marginally over time in both groups (Friedman *p* = 0.046 and *p* = 0.023), with no significant between–group difference at any time point. HIZ decreased significantly over time in both groups (both *p* < 0.001) without between–group differences. Nerve–root compression decreased significantly over time in both groups (both *p* < 0.001); the between–group difference was significant only at 3 months (*p* = 0.045). Modic–category distribution did not differ significantly between groups at any time point (all *p* > 0.4). Representative pre– and post–treatment lumbosacral MRI images are shown for one patient treated with ACS (Figure 3) and one patient treated with microdiscectomy (Figure 4).

**Figure 3:**
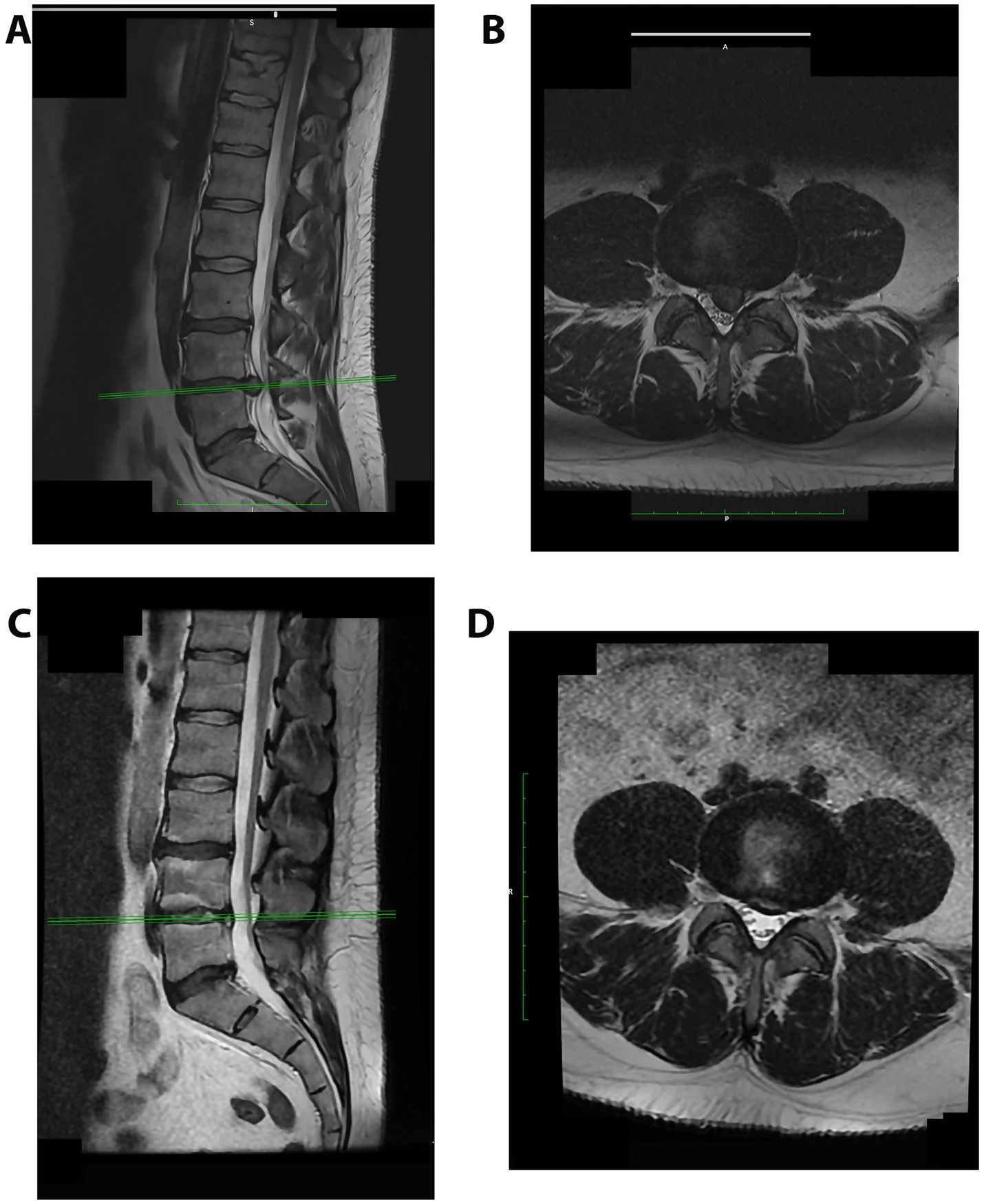
Representative lumbosacral MRI of a patient treated with autologous conditioned serum (ACS, Orthokine®) before and after treatment. **(A)** Sagittal T2–weighted image before treatment. **(B)** Axial T2–weighted image at the corresponding level before treatment. **(C)** Sagittal T2–weighted image after treatment. **(D)** Axial T2–weighted image at the corresponding level after treatment.

**Figure 4:**
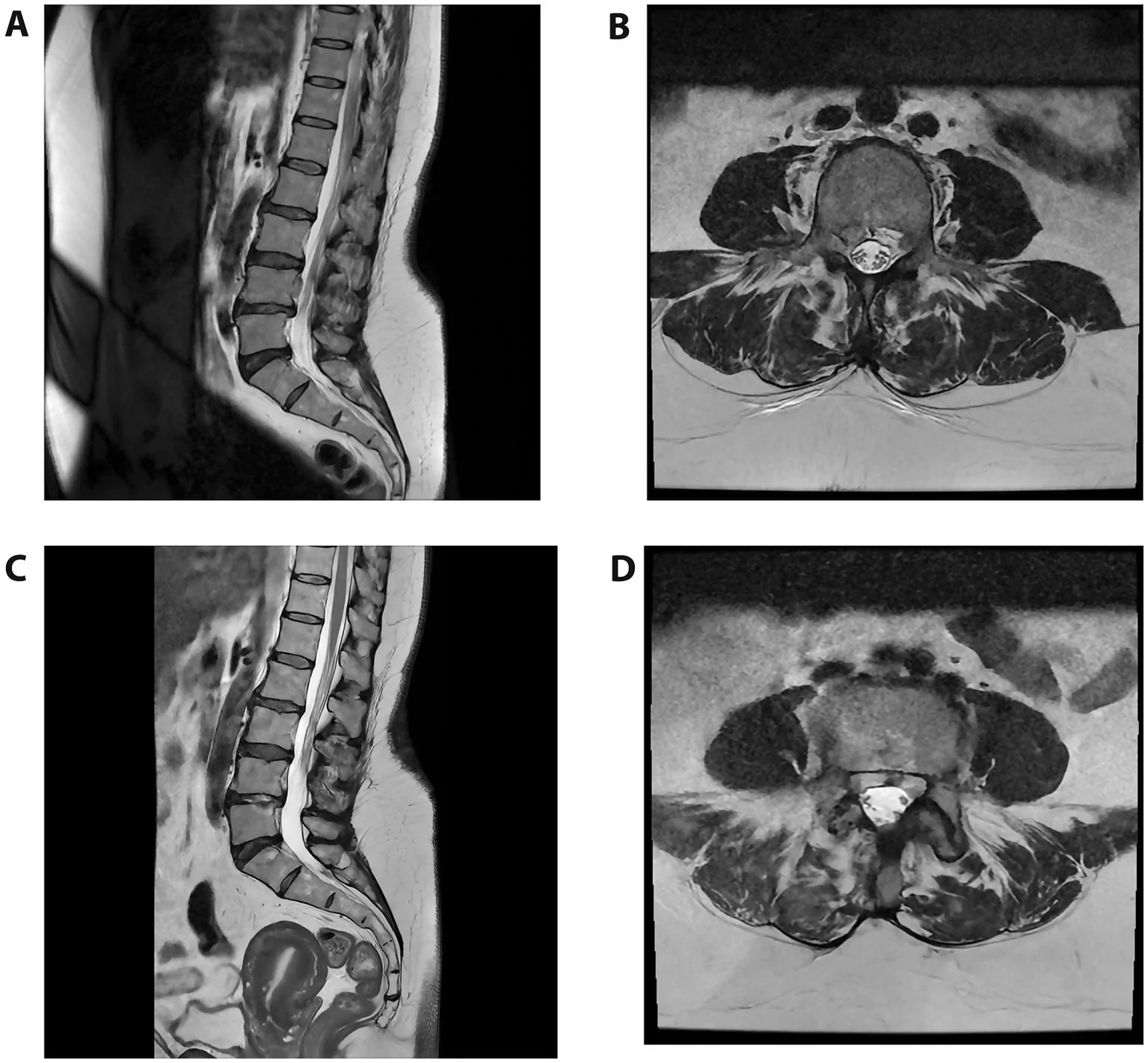
Representative lumbosacral MRI of a patient treated with microdiscectomy before and after treatment. **(A)** Sagittal T2–weighted image before treatment. **(B)** Axial T2–weighted image at the corresponding level before treatment. **(C)** Sagittal T2–weighted image after treatment. **(D)** Axial T2–weighted image at the corresponding level after treatment.

Detailed per–domain SF–36 results, full DSSS subscale data, the sex–stratified DSSS analysis, and the complete sensitivity (ANCOVA) analysis are provided in the Supplementary Figures S1–S3 and Supplementary Tables S1–S4.

## 4 Discussion

In this prospective cohort of patients with lumbar DDD and radiculopathy refractory to conservative treatment, both microdiscectomy and ACS produced large, clinically important improvements across pain, disability, pain quality, HRQoL, life satisfaction, fatigue, and sexual satisfaction, sustained throughout the 12–month observation period. The consistent pattern was an early microdiscectomy advantage, apparent mainly at 3 months, followed by convergence by 12 months. To our knowledge, this is among the first studies to compare a surgical and a biologically oriented treatment for lumbar DDD using a validated, multidimensional sexual–satisfaction instrument alongside conventional outcomes.

The early microdiscectomy advantage in pain, disability, pain quality, and HRQoL fits the mechanistic distinction between treatments: microdiscectomy achieves immediate mechanical decompression, whereas ACS acts more gradually by modulating the IL–1–driven inflammatory cascade implicated in disc degeneration and radicular pain (9). The convergence of both groups by 12 months suggests that once the inflammatory and mechanical drivers of radicular pain are adequately addressed by either route, both treatments reach a broadly similar clinical end–state within the first year.

An unexpected, mechanistically coherent finding was the greater increase in disc height in the ACS group from 6 months onward, with the largest group × time interaction effect size observed (η^2^p = 0.29). A greater increase in measured disc height after orthobiologic treatment was previously reported mainly in preclinical and platelet–rich plasma models (13, 14); our findings extend this observation to ACS in a clinical cohort. This warrants cautious interpretation given the observational design and reliance on a simple morphometric parameter rather than compositional imaging; T2 relaxometry mapping could clarify whether this reflects genuine matrix regeneration rather than reduced inflammation or altered loading (3). If confirmed, this would suggest ACS offers a disease–modifying, not purely symptomatic, benefit—meriting dedicated, blinded imaging–based confirmation in future trials.

Sexual dysfunction is a common, multidimensional consequence of chronic musculoskeletal pain (4, 5, 15, 16). The ODI’s single sexual–activity item cannot capture the physical, emotional, and control–related dimensions assessed by the DSSS. Both groups improved from a moderate baseline to near–maximal scores at 12 months, mirroring pain and disability trajectories—consistent with models in which sexual functioning is substantially mediated by pain, fear of movement, and disability rather than an independent domain (15, 16, 30, 31).

The subscale–level results add nuance: microdiscectomy showed an early (3–month) advantage across all three DSSS subscales, whereas by 12 months ACS showed a significant advantage on the Physical subscale and a borderline advantage on the Emotional subscale, with no difference on Control. Plausible explanations include the absence of a surgical wound in the ACS group and its greater disc–height recovery (Section 4.3); given the modest subgroup size, a chance finding cannot be excluded. The Control subscale—reflecting perceived agency over one’s sexual life rather than the physical experience of intercourse—may be less sensitive to treatment modality and more dependent on broader psychological adaptation (16).

Unlike pain– and disability–related measures, SWLS and FACIT–F showed no significant group × time interaction, improving in parallel regardless of modality—consistent with a biopsychosocial view in which life satisfaction and fatigue are downstream consequences of overall pain and disability burden rather than domains differentially modulated by treatment (30, 31). Global well–being thus appears to improve along a common trajectory once effective pain relief is achieved, consistent with prior work linking fatigue to pain intensity and disability (32).

Patients differed modestly in age and, more substantially, in herniation morphology (more extrusions/sequestrations with microdiscectomy, more contained protrusions with ACS), reflecting routine clinical decision–making—a textbook example of confounding by indication in non–randomised comparative–effectiveness research (33). Our ANCOVA sensitivity analysis reproduced the same overall pattern as the unadjusted analysis (Section 4.2.5), increasing confidence that these findings are not fully explained by baseline imbalances alone, though unmeasured confounding cannot be excluded.

Strengths include a comprehensive outcome battery—pain, disability, pain quality, physical and mental HRQoL, life satisfaction, fatigue, and a validated three–subscale sexual–satisfaction instrument—combined with radiological follow–up at every time point. Limitations include non–randomised allocation, producing differences in herniation morphology and age between groups (sensitivity analyses confirmed the main findings, though residual confounding cannot be excluded); reliance on conventional MRI disc–height measurement rather than compositional imaging; and a 12–month follow–up that cannot establish long–term durability. While a longer follow-up would help characterise durability and late recurrence, treatment-effect differences after lumbar disc surgery have been shown to largely stabilise within the first year and persist over several years (34), consistent with the 12-month endpoint commonly used to define surgical success in outcome registries (35); some recurrence of symptoms beyond 1 year has nonetheless been reported (36). As an early, exploratory study with a relatively short follow-up and limited sample size, these findings should not be taken to support causal or broadly generalizable conclusions, given the heterogeneous patient population, unequal group sizes, and lack of stratification by radiculopathy severity. Given the broad outcome battery and the number of time points examined, corrections for multiplicity were not applied, and secondary and subgroup comparisons should be regarded as hypothesis–generating. The relatively small ACS subgroup (*n* = 54), and the correspondingly smaller sex–stratified and subscale–level analyses, may have limited statistical power to detect modest between–group or between–sex differences. Restricting enrolment to a single self–reported ethnicity (White/Caucasian) reduced variability related to population–level differences in disc biology and pain reporting, strengthening internal comparability between the two treatment groups, but it also means that generalisability of these findings to other populations remains to be confirmed. Serum CRP and the sex–stratified DSSS analysis were exploratory secondary measures rather than *a priori* primary outcomes and should be interpreted accordingly. Concomitant analgesic use and physiotherapy during follow–up were not systematically recorded in either group and could not be formally adjusted for; all 140 patients nonetheless completed the assigned treatment course with data at every time point, and no patient for whom ACS was recommended deteriorated to the point of crossing over to surgery. The comparatively greater increase in disc height and canal occupation after ACS should also be weighed against the fact that, in the microdiscectomy group, part of the herniated disc material was surgically removed. Both groups were assessed using the same MRI protocol and independent readings by the same panel; however, blinding to the subsequently selected treatment pathway applied only to the baseline assessment, because postoperative changes could reveal treatment allocation on follow–up images.

Overall, outcomes did not differ significantly between ACS and microdiscectomy by 12 months across pain, disability, HRQoL, fatigue, and sexual satisfaction, while ACS offered a less invasive alternative, consistent with the growing orthobiologic literature on IL–1–targeted therapies for discogenic pain. Relief developed more gradually after ACS, but both treatments produced substantial, meaningful improvement by study end.

These findings suggest that treatment selection between microdiscectomy and ACS should weigh not only the expected speed of relief but also patient preference, invasiveness, and broader quality–of–life goals. Microdiscectomy remains preferred when rapid decompression is clinically required, while ACS may offer an attractive, less invasive alternative for selected patients able to tolerate a more gradual response. The greater disc–height recovery after ACS, together with its favourable late–stage sexual–satisfaction profile, warrants dedicated mechanistic study using compositional MRI such as T2 mapping or MR elastography. Future randomised trials should incorporate this multidimensional outcome battery—including validated sexual–satisfaction and fatigue instruments—with longer follow–up, to confirm comparative effectiveness and durability beyond 12 months. Given that several radiological parameters (e.g., nerve–root compression) improved over time in both groups, ideally such trials would also incorporate a conservative–treatment (no active intervention) comparison arm. This would help distinguish treatment–specific effects from the natural history of contained herniations.

## Data Availability

The original contributions presented in the study are included in the article/Supplementary material, further inquiries can be directed to the corresponding author.
