# Supplementary material for: Microdiscectomy versus autologous conditioned serum for degenerative lumbar disc disease with radiculopathy: a 12–month exploratory study
Source: Front Neurol. 2026 Oct 20;17:1972710. doi: 10.3389/fneur.2026.1972710 (PMC13727616; doi:10.3389/fneur.2026.1972710)
Supplement: Supplementary file 1 [file Supplementary_file_1.docx]

Supplementary Material

# 1 Supplementary Data

*Figure S1. (A) Disability (Oswestry Disability Index, ODI); (B) pain quality (SF-MPQ total score) over time by treatment group.*

*Figure S2. SF-36 (A) Physical Component Summary (PCS) and (B) Mental Component Summary (MCS) scores over time by treatment group.*

*Figure S3. (A) Life satisfaction (SWLS); (B) fatigue (FACIT-F); (C) sexual satisfaction (DSSS total score) over time by treatment group.*

*Table S1. Detailed per-domain SF-36 scores (mean ± SD) over time by treatment group. * p < 0.05 for the between-group comparison at that time point (Welch t-test).*

| **SF-36 domain** | **Time point** | **Microdiscectomy (n = 86)** | **ACS (n = 54)** | **p*** |
| --- | --- | --- | --- | --- |
| Physical Functioning (0–100) | T0 | 23.68 ± 9.98 | 21.12 ± 10.30 | 0.150 |
|  | T3 | 63.99 ± 10.59 | 58.94 ± 11.09 | 0.009 * |
|  | T6 | 72.60 ± 10.88 | 69.41 ± 10.58 | 0.088 |
|  | T12 | 75.70 ± 11.44 | 76.14 ± 11.49 | 0.826 |
| Role-Physical (0–100) | T0 | 24.45 ± 10.03 | 22.90 ± 10.95 | 0.404 |
|  | T3 | 63.80 ± 12.07 | 57.34 ± 11.73 | 0.002 * |
|  | T6 | 72.98 ± 12.09 | 70.02 ± 12.95 | 0.180 |
|  | T12 | 75.72 ± 11.48 | 75.65 ± 11.19 | 0.975 |
| Bodily Pain (0–100) | T0 | 18.73 ± 8.26 | 17.54 ± 10.68 | 0.484 |
|  | T3 | 67.88 ± 10.18 | 60.69 ± 11.25 | <0.001 * |
|  | T6 | 78.29 ± 12.62 | 74.60 ± 11.57 | 0.078 |
|  | T12 | 82.87 ± 13.66 | 83.24 ± 11.50 | 0.865 |
| General Health (0–100) | T0 | 40.72 ± 7.90 | 37.72 ± 8.31 | 0.036 * |
|  | T3 | 66.88 ± 7.27 | 64.17 ± 7.94 | 0.045 * |
|  | T6 | 74.35 ± 7.91 | 72.34 ± 7.32 | 0.129 |
|  | T12 | 77.16 ± 8.56 | 77.57 ± 8.91 | 0.788 |
| Vitality (0–100) | T0 | 33.81 ± 8.04 | 32.41 ± 8.80 | 0.348 |
|  | T3 | 64.86 ± 8.21 | 61.33 ± 9.08 | 0.022 * |
|  | T6 | 71.39 ± 8.75 | 69.99 ± 8.66 | 0.357 |
|  | T12 | 73.97 ± 9.77 | 75.37 ± 8.62 | 0.378 |
| Social Functioning (0–100) | T0 | 35.86 ± 8.67 | 35.42 ± 9.46 | 0.785 |
|  | T3 | 68.46 ± 8.12 | 64.38 ± 12.14 | 0.032 * |
|  | T6 | 74.56 ± 10.43 | 73.50 ± 9.80 | 0.544 |
|  | T12 | 77.41 ± 9.63 | 78.57 ± 11.36 | 0.534 |
| Role-Emotional (0–100) | T0 | 38.72 ± 11.64 | 38.10 ± 10.34 | 0.744 |
|  | T3 | 66.79 ± 9.01 | 64.42 ± 11.01 | 0.188 |
|  | T6 | 75.10 ± 10.55 | 73.45 ± 9.47 | 0.340 |
|  | T12 | 76.37 ± 11.81 | 78.20 ± 10.62 | 0.343 |
| Mental Health (0–100) | T0 | 42.51 ± 6.47 | 43.34 ± 6.63 | 0.472 |
|  | T3 | 69.07 ± 7.49 | 65.87 ± 8.35 | 0.024 * |
|  | T6 | 74.79 ± 7.60 | 73.99 ± 7.03 | 0.528 |
|  | T12 | 77.28 ± 7.82 | 77.39 ± 8.12 | 0.933 |

*Table S2. DSSS subscale scores over time by treatment group. * p < 0.05 for the between-group comparison at that time point.*

| **DSSS subscale (range)** | **Time point** | **Microdiscectomy (n = 86)** | **ACS (n = 54)** | **p*** |
| --- | --- | --- | --- | --- |
| **Physical (11–55)** | T0 | 25.84 ± 3.93 | 27.16 ± 4.07 | 0.061 |
|  | T3 | 38.54 ± 4.11 | 36.51 ± 4.59 | **0.009 *** |
|  | T6 | 41.30 ± 4.42 | 41.74 ± 3.86 | 0.544 |
|  | T12 | 42.19 ± 3.87 | 43.52 ± 4.12 | **0.024 *** |
| **Emotional (4–20)** | T0 | 9.24 ± 1.85 | 9.98 ± 1.75 | **0.015 *** |
|  | T3 | 14.03 ± 1.78 | 13.38 ± 1.78 | **0.033 *** |
|  | T6 | 14.58 ± 2.02 | 15.10 ± 1.53 | 0.086 |
|  | T12 | 15.31 ± 1.88 | 15.90 ± 1.84 | 0.055 |
| **Control (6–30)** | T0 | 14.07 ± 2.32 | 14.44 ± 2.14 | 0.338 |
|  | T3 | 20.87 ± 2.46 | 19.93 ± 2.40 | **0.027 *** |
|  | T6 | 22.62 ± 2.54 | 22.77 ± 2.57 | 0.763 |
|  | T12 | 23.31 ± 2.43 | 23.66 ± 2.55 | 0.420 |

Values are mean ± SD.

*Table S3. Davies Sexual Satisfaction Scale (DSSS) total score over time, stratified by sex, within each treatment group.*

| **Group** | **Time point** | **Male (mean ± SD)** | **Female (mean ± SD)** | **p*** |
| --- | --- | --- | --- | --- |
| Microdiscectomy (n = 86: 54 M / 32 F) | T0 | 49.48 ± 7.25 | 48.56 ± 7.05 | 0.566 |
|  | T3 | 73.95 ± 6.99 | 72.64 ± 7.86 | 0.441 |
|  | T6 | 79.18 ± 7.78 | 77.43 ± 8.92 | 0.361 |
|  | T12 | 81.62 ± 7.86 | 79.38 ± 5.84 | 0.136 |
| ACS / Orthokine® (n = 54: 32 M / 22 F) | T0 | 51.34 ± 7.25 | 51.93 ± 6.41 | 0.756 |
|  | T3 | 71.52 ± 7.96 | 67.35 ± 7.26 | 0.052 |
|  | T6 | 80.83 ± 7.32 | 77.78 ± 6.50 | 0.114 |
|  | T12 | 85.07 ± 7.68 | 80.16 ± 7.04 | 0.019 * |

*Values are mean ± SD. *Between-sex comparison at each time point within the treatment group (Welch t-test). Mixed-design (sex × time) ANOVA within each group: microdiscectomy – sex main effect F(1,84) = 1.88, p = 0.174; sex × time interaction F(3,252) = 0.16, p = 0.921; ACS – sex main effect F(1,52) = 3.83, p = 0.056; sex × time interaction F(3,156) = 2.38, p = 0.072.*

*Table S4. ANCOVA sensitivity analysis for the principal pain and disability outcomes (adjusted for baseline value, age, and baseline herniation type), compared with the unadjusted between-group p-value.*

| **Outcome** | **Time point** | **Unadjusted p** | **Adjusted mean difference (95% CI)*** | **ANCOVA p-value*** |
| --- | --- | --- | --- | --- |
| VAS (0-10) | 3 months | <0.001 * | -0.58 (-0.94 to -0.22) | 0.002 * |
| VAS (0-10) | 12 months | 0.810 | 0.11 (-0.37 to 0.58) | 0.654 |
| NRS (0-10) | 3 months | <0.001 * | -0.67 (-1.07 to -0.26) | 0.001 * |
| NRS (0-10) | 12 months | 0.674 | -0.05 (-0.54 to 0.44) | 0.846 |
| ODI (%) | 3 months | 0.002 * | -4.37 (-7.39 to -1.34) | 0.005 * |
| ODI (%) | 12 months | 0.616 | -0.86 (-4.63 to 2.91) | 0.654 |

Values are microdiscectomy minus ACS. * From ANCOVA model: outcome ~ group + baseline value + age + herniation type (extrusion / protrusion / sequestration). Unadjusted p reproduces the same between-group test reported in the Results and Table 2 (Welch t-test where normality was approximately met in both groups; Mann-Whitney U otherwise, as was the case for these outcomes at these time points), shown here for direct comparison with the ANCOVA-adjusted estimate.
